# Genomic alterations in cholangiocarcinoma: clinical significance and relevance to therapy

**DOI:** 10.37349/etat.2022.00079

**Published:** 2022-04-26

**Authors:** Marianeve Carotenuto, Alessandra Sacco, Laura Forgione, Nicola Normanno

**Affiliations:** Cell Biology and Biotherapy Unit, Istituto Nazionale Tumori-IRCCS-Fondazione G. Pascale, 80131 Naples, Italy; University of Southampton, UK

**Keywords:** Cholangiocarcinoma, molecular profiling, genomic alterations, targeted therapy, circulating tumor DNA

## Abstract

Improving the survival of patients with cholangiocarcinoma (CCA) has long proved challenging, although the treatment of this disease nowadays is on advancement. The historical invariability of survival outcomes and the limited number of agents known to be effective in the treatment of this disease has increased the number of studies designed to identify genetic targetable hits that can be efficacious for novel therapies. In this respect, the increasing feasibility of molecular profiling starting either from tumor tissue or circulating cell-free DNA (cfDNA) has led to an increased understanding of CCA biology. Intrahepatic CCA (iCCA) and extrahepatic CCA (eCCA) display different and typical patterns of actionable genomic alterations, which offer opportunity for therapeutic intervention. This review article will summarize the current knowledge on the genomic alterations of iCCA and eCCA, provide information on the main technologies for genomic profiling using either tumor tissue or cfDNA, and briefly discuss the main clinical trials with targeted agents in this disease.

## Introduction

Cholangiocarcinoma (CCA) is a rare malignancy arising from the epithelium of the bile ducts, belonging to the group of biliary tract cancers (BTCs), along with gallbladder and ampullary carcinomas. CCA is the second most common primary hepatic malignancy, after hepatocellular carcinoma, and accounts for approximately 10–20% of all hepatobiliary malignancies [1].

Risk factors for CCA include primary sclerosing cholangitis, liver fluke infestation, bile duct anomalies, biliary papillomatosis, chemical carcinogens including thorotrast and nitrosamines, obesity, nonalcoholic liver disease, and viral hepatitis [2–5].

Based on anatomical location of the primary tumor, CCA is classified into intrahepatic (iCCA) and extrahepatic (eCCA), and eCCA is further classified as perihilar (pCCA) or distal CCA (dCCA). iCCA is the second most common primary malignant liver tumor accounting for 10–15% of hepatobiliary neoplasm [6]. It arises from the intrahepatic bile ducts of hepatic parenchyma and represents the 10–20% of all CCA. Based on the level or size of the affected duct, iCCA has two main variants. CCA and small duct type iCCA occur in small intrahepatic bile ducts, and hepatic stem or progenitor cells (HpSCs) and cuboidal cholangiocytes are the putative cells of origin of these malignancies, respectively [7]. In contrast, large bile duct iCCA arises in large intrahepatic bile ducts and likely originates from columnar cholangiocytes or peribiliary glands [7]. However, it must be emphasized that controversies exist regarding the cellular origins of iCCA based on lineage tracing studies in experimental carcinogenetic models, which provided evidence in favour of HpSC, cholangiocyte, or hepatocyte origin of iCCA [8, 9].

eCCA is an epithelial cancer that develops in the bile ducts outside of the liver and exhibits features of cholangiocytic differentiation. The two eCCA subtypes (pCCA and dCCA) arise from mucosal columnar cholangiocytes or peribiliary glands, which are also implicated in the origin of precursor lesions [7]. Although most of the cells contained in the peribiliary glands are mature epithelial cells, few populations have been identified expressing immature stem or progenitor cell markers and phenotypes. These cells have been shown to proliferate in response to bile duct injury, thus functioning as a niche for bile stem cells and representing the potential cell of origin of eCCA [10–13].

These subtypes not only differ in their anatomical location but also in their etiopathogenesis; indeed, they are characterized by distinct risk factors, different proposed cells of origin, and their genomic aberrations in particuar [14–18]. In particular, pCCA is the most common subtype of CCA accounting for 60% of biliary tract tumor and is located in the right and/or left hepatic duct and/or at their junction; dCCA involves the mid or lower half of the bile duct and accounts for approximately 20–30% of CCA [19].

Based on histologic criteria, CCAs can be classified as well, moderately, or poorly differentiated adenocarcinomas, or rare variants [20]. However, these criteria have proven to be insufficient tools for guiding treatment decisions to improve outcomes.

Epidemiological studies suggest that rates of iCCA are increasing, particularly in Western countries; conversely, the incidence of both pCCA and dCCA appears to be declining [4].

Worldwide, the average age at diagnosis is > 50 years, except for patients with primary sclerosing cholangitis [21]. Furthermore, CCA is slightly more common and mortality is higher in males than females [22].

CCA is characterized by a poor overall prognosis and median overall survival (mOS) less than two years in patients with advanced disease. Due to pathologic heterogeneity and lack of specific symptoms, CCA is rarely diagnosed at early stages. Surgical resection potentially represents the best curative treatment option although is limited to patients diagnosed in the early stages. Patients with inoperable disease have median survival that is approximately 6 months and less than one year, respectively for iCCA and eCCA [23]. Moreover, the cancer recurrence rate is high even after surgical resection and the addition of adjuvant chemotherapy after surgical resection did not improve the overall survival, as was expected [24].

In patients with advanced disease, the combination of gemcitabine and cisplatin still represents the only standard first line treatment option. The phase 3 ABC-06 study demonstrated the activity of folinic acid, fluorouracil and oxaliplatin (FOLFOX) as second line treatment. Recently, genetic profiling studies have highlighted the genetic diversity between subtypes and the genomic complexity of CCA, which offers possibility for therapeutic intervention with target therapies matching specific genomic alterations. In this review article, the current knowledge about genomic alterations typical of each subtype of CCA will be summarized, outlining unresolved areas of debate that warrant further study.

## The landscape of genomic alterations across CCA subtypes

Broad implementation of comprehensive genomic profiling in daily clinical screening, thanks to the development of next-generation sequencing (NGS) technology, allowed a better understanding of the molecular mechanisms occurring in CCA. The frequency of actionable genomic alterations identified in CCA and their associations with the anatomical subtypes are summarized in Table 1 and described below.

**Table 1.** Actionable genomic alterations in iCCA and associations with anatomic sub-type

**CCA sub-type**	**Targeted gene**	**Frequency**	**References**
iCCA	IDH1/2	10–20%	[26–28]
	FGFR	7–16%	[26, 27, 47, 50]
	BRAF V600E	5%	[26, 27, 71–75, 79]
	KRAS	8–54%	[75]
	ERBB2	8%	[26, 27]
	PI3K	7%	[26, 27]
	NTRK	≤ 1%	[84]
	BRCA1	0.4%	[92]
	BRCA2	2.7%	
eCCA	KRAS	43%	[92]
	ERBB2	5–9%	[90, 94]
	PI3K	5%	[94]
	IDH1/2	2–3%	[94]
	EGFR	1%	[94]
	BRCA1	0.4%	[94]
	BRCA2	2.7%	

IDH1/2: isocitrate dehydrogenase (NADP^+^) 1/2; FGFR: fibroblast growth factor receptor; BRAF: B-Raf proto-oncogene; KRAS: KRAS proto-oncogene; ERBB2: erb-b2 receptor tyrosine kinase 2; NTRK: neurotrophic receptor tyrosine kinase

### iCCA

**Figure 1.**
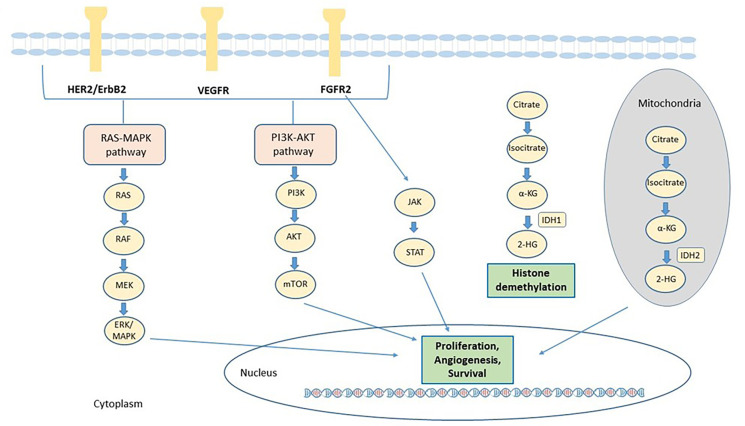
Main actionable signaling pathways in CCA. The signaling pathways involved in CCA progression and for which targeted agents are available, include receptor tyrosine kinases such as FGFR2 and ERBB and downstream signaling proteins, such as Janus kinases (JAK)/signal transducer and activator of transcription proteins (STAT), rat sarcoma virus (RAS)/Raf-1 proto-oncogene, serine/threonine kinase (RAF)/mitogen-activated extracellular signal-regulated kinases (ERK) kinase (MEK)/(ERK) and phosphatidylinositol 3-Kinase (PI3K). In addition, mutant IDH enzymes acquire the capacity to synthesize 2-HG from α-ketoglutarate (α-KG). 2-HG alters the activity of α-KG–dependent dioxygenase enzymes involved in cell differentiation, survival, and DNA methylation. HER2: human epidermal growth factor receptor 2; VEGFR: vascular endothelial growth factor receptor; MAPK: mitogen-activated protein kinase; AKT: serine/threonine-protein kinase; mTOR: mammalian target of rapamycin

In the recent years, several efforts have been made to identify the genetic alterations associated to iCCA, to improve the understanding of its genomic complexity. The prevalence of mutation has been found quite variable across different studies, probably due to tumor heterogeneity, to the relative rarity of these samples compared to the other tumors and the different sequencing technique used. Many genetic alterations in oncogenes and tumor suppressor genes were identified in iCCA, including epidermal growth factor receptor (*EGFR*), *KRAS*, *BRAF*, tumor protein p53 (*TP53*), BReast CAncer gene 1 (*BRCA1*) associated protein 1 (*BAP1*), *IDH1*, *IDH2*, *FGFR2*, ROS proto-oncogene 1, receptor tyrosine kinase (*ROS1*), polybromo 1 (*PBRM1*) and AT-rich interaction domain 1A (*ARID1A*). The main signaling pathways involved in the pathogenesis of CCA and for which targeted agents are available, are described in Figure 1.

Results from large genomic profiling studies suggest that *IDH1* and *FGFR2* are the most common genes with actionable alterations in iCCA. Based on the FIGHT-202 study, 45% of the patients analysed had clinically actionable genetic alterations: IDH1 missense mutations (10%), alterations in *ERBB2* (8%), *FGFR2* (7%), phosphatidylinositol-4,5-bisphosphate 3-kinase catalytic subunit alpha (*PIK3CA*, 7%) and *BRAF* (5%) genes [25]. Similar results were obtained in the studies by Javle et al. [26] and by Lowery et al. [27], who profiled respectively 4,000 and 195 CCA samples.

#### IDH mutations

Mutations in *IDH1/2* have been reported in 10–20% of iCCA cases; in particular, the occurrence of *IDH1* and *IDH2* has been reported in 7–20% and 3% of iCCA, respectively [28]. *IDH* and *FGFR* alterations are mainly mutually exclusive [29], although co-occurring IDH1 mutations were found in 5.1% of patients with *FGFR2*-rearranged iCCA [25].

Gain-of-function *IDH* variants lead to the production of the oncometabolite *d*-2-hydroxyglutarate (2-HG), whose accumulation is implicated in epigenetic alterations and impaired cellular differentiation [18]. In particular, high levels of 2-HG are associated with increased methylation of the cytosine-phosphate-guanine (CpG) sites and altered histone methylation that cause a block of cellular differentiation and induce tumorigenesis [30]. Moreover, *IDH* mutations cause alterations in hypoxia signaling and collagen processing, and activation of epithelial-to-mesenchymal transition (EMT) via increased expression of Zinc Finger E-Box Binding Homeobox 1 (*ZEB1*) and decreased levels of microRNA-200. In addition, *IDH* mutations often interact with tyrosine kinase and MAPK-dependent signaling pathways [30].

*IDH1* hotspots are located in the arginine 132 residue, exactly IDH1-R132C (44%) and IDH1-R132G (14%). Moreover, Grassian and colleagues [30] reported that *IDH1* and *IDH2* mutations are also mutually exclusive with NRAS proto-oncogene (*NRAS*)/*KRAS* alterations. *IDH1* mutations may co-exist with *ARID1A* (22.0%), *BAP1* (15.5%) and *PBRM1* mutations or loss (13.3%) [31].

The relationships of *IDH* mutations with prognosis and clinicopathologic features remain still controversial in CCA [32]. While some studies showed that *IDH* mutations were associated with poorly differentiated CCA and clear-cell histology, others showed no association with histological grade. Significant differences in *IDH* mutations between certain types of parasite-associated CCA have also been reported [33]. Several studies investigated the prognostic significance of *IDH* mutation in patients with iCCA, although none of them reported a statistically significant association between the presence of *IDH1* mutations and clinical outcomes [29, 32, 34–40].

#### FGFR genomic alterations

*FGFR* fusion genes have been reported in a wide range of cancers, including iCCA [41].

*FGFR* fusions can be classified in two types. In type 1 fusions, the extracellular and the transmembrane part of the receptors are replaced by the fusion partners, which includes only the FGFR kinase domain linked to the 5’ protein partner. While in type 2 fusion, the breakpoint usually occurs in exons 17, 18, or 19, and the resulting fusion proteins, are transmembrane-type FGFRs with C-terminal substitution to the region of fusion partners [42].

In both types of fusion protein, the different *FGFR* fusion partners contribute with specific domains to facilitate the dimerization [43] and such ligand-independent dimerization provides oncogenic potential to the FGFR fusion protein. Dysregulation of FGFR signaling leads to an antiapoptotic, mutagenic and angiogenic response in cells, through the activation of the RAS/MAPK, PIK3CA/AKT and STAT1 pathways [44, 45].

*FGFR* fusions together with FGFR mutations are rather rare, suggesting that the presence of unique alterations is sufficient to drive cancer progression [41].

*FGFR2* gene is located on chromosome 10, and around 50% of FGFR2-fusions evolve through intrachromosomal events [46]. *FGFR2* fusions are particularly common in CCA, and over 100 different *FGFR2* fusion partners have been reported in this disease [27, 47–52].

**Figure 2.**
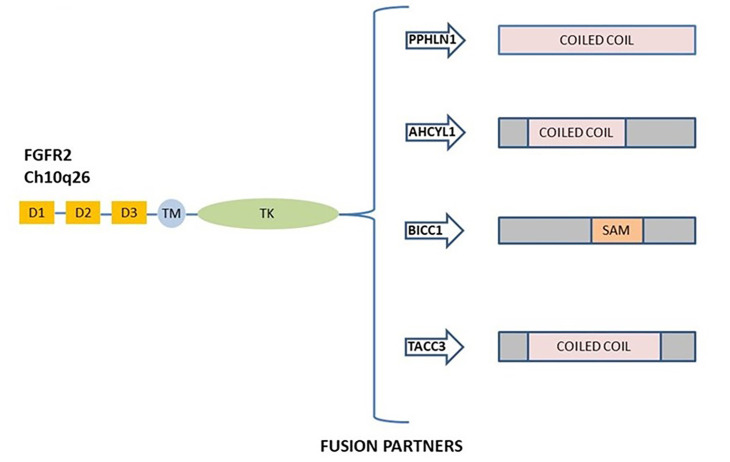
Schematic representation of commonly *FGFR2* gene fusion in ICC. The most frequent rearrangement partners FGFR2–PPHLN1, FGFR2–AHCYL1, FGFR2–BICC1, and FGFR2–TACC3 are all shown. D: domain; TM: transmembrane region; TK: tyrosine kinase domain; SAM: sterile alpha motif domain

*FGFR2* fusions derived by chromosomal events activate the canonical FGFR signaling and possess oncogenic activity [51, 53]. *FGFR2* gene fusions were observed in 10–16% of patients with iCCA [47, 50], and the most prevalent fusions described are FGFR2–adenosylhomocysteinase like 1 (*AHCYL1*), FGFR2–BicC family RNA binding protein 1 (*BICC1*), FGFR2–periphilin 1 (*PPHLN1*), and FGFR2–transforming acidic coiled-coil containing protein 3 (*TACC3*) [54]. A schematic representation of the most common *FGFR2* gene fusions in iCCA is shown in Figure 2.

In studies screening both iCCA and eCCA, *FGFR2* fusions were detected exclusively in patients with iCCA (13.6%) and were found mutually exclusive with *KRAS*/*BRAF* mutations [18, 47].

Other less frequent rearrangement partners of *FGFR2* described in iCCA are *O*-GlcNAcase (*OGA*) [50], shootin 1 (*SHTN1*), DExD-box helicase 21 (*DDX21*), laminin subunit gamma 1 (*LAMC1*), nebulin related anchoring protein (*NRAP*), nucleolar protein 4 (*NOL4*), polyhomeotic homolog 1 (*PHC1*), RAB guanosine triphosphate hydrolase (GTPase) activating protein 1 like (RABGAP1L), RAS protein activator like 2 (*RASAL2*), Rho associated coiled-coil containing protein kinase 1 (*ROCK1*), transcription factor EC (*TFEC*), AF4/FMR2 family member 4 (*AFF4*), CUGBP Elav-like family member 2 (*CELF2*), dynactin subunit 2 (*DCTN2*), DnaJ heat shock protein family (Hsp40) member C12 (*DNAJC12*), DAZ interacting zinc finger protein 1 (*DZIP1*), forkhead box P1 (*FOXP1*), internexin neuronal intermediate filament protein alpha (*INA*), potassium channel tetramerization domain containing 1 (*KCTD1*), lengsin (*LGSN*), leupaxin (*LPXN*), myopalladin (*MYPN*), parkin RBR E3 ubiquitin protein ligase (*PRKN*), pericentriolar material 1 (*PCM1*), ring finger protein 41 (*RNF41*), SH3 domain containing GRB2 like, endophilin B1 (*SH3GLB1*), serine/threonine kinase 3 (*STK3*), sorbin and SH3 domain containing 1 (*SORBS1*), TBC1 domain family member 1 (*TBC1D1*) and ubiquilin 1 (*UBQLN1*) [27, 48, 49]. However, the biological activity of these fusions has not been fully clarified.

Some studies suggested a possible correlation between *FGFR* fusions and a better prognosis in iCCA patients. Graham and colleagues [55] evaluated the presence of *FGFR2* translocations by fluorescence in situ hybridization (FISH) analysis in 152 CCA and 4 intraductal papillary neoplasms of the bile duct. Among these, 30 patients were found positive for *FGFR2* translocations. The study reported that the median cancer-specific survival interval for FGFR2-rearranged patients was significantly longer (123 months) compared to that for patients without FGFR2 translocations (37 months, *P* = 0.039) [55].

Such correlation was confirmed in an additional study where 377 patients with BTC were screened and 95 FGFR genetic alterations were detected, including 63 *FGFR2* fusions. Patients carrying *FGFR* alterations had significantly longer overall survival (OS) than patients without FGFR aberrations (37 *vs.* 20 months; *P* < 0.001) [49]. Although the difference was not statistically significant, it was reported that in patients with fluke associated-iCCA, the presence of rare fusions of *FGFR2* correlated with better OS than that of fusion-negative tumors [56]. Moreover, the presence of *FGFR2* genetic alterations, which occurs more frequently in younger patients (≤ 40 years; 20%) is associated with indolent disease progression and a better prognosis [49].

In the study by Silverman et al. [25], no patient with FGFR2 rearrangement was found to have high tumor mutation burden (TMB) or microsatellite instability (MSI). MSI is due to either germline or somatic mutations of the genes involved in the mismatch repair (MMR) mechanism of DNA repair. In this respect, the lifetime risk for bile duct cancer in patients with Lynch syndrome is approximately 2% [57]. Notably, data about the prevalence of MSI in CCA are conflicting. Nakamura and colleagues [18] reported that approximately 6% of biliary cancers are hypermutated, including 2% with MMR deficiency. Several recent studies suggest that MSI occurs rarely in western CCA patients [58–60].

#### Additional genetic alterations of iCCA

Members of the ERBB tyrosine kinase family present some of the most commonly altered proteins in cancer. The aberrant regulation of ERBB2 and the EGFR signaling plays an important role in the pathogenesis of iCCA. Increased expression and DNA amplification of *ERBB2* was reported in 0 to 73% of iCCA [61, 62]. However, no mutation in *ERBB2* has been detected nowadays [61, 63–70].

Mutations in *EGFR* as well as hotspot mutations in its downstream genes, such as *PIK3CA* (9–32%) and *BRAF* (0–22%) with V600E, the most common *BRAF* mutation, are rare events in iCCA and occurs in approximately 5% of patients [71–75], but is now being shown to have some independent prognostic significance. Activating mutations of *BRAF*, such as the V600E, lead to the constitutive dimerization of the BRAF protein, which in turn activates the RAF/MEK/ERK signaling cascade, thus promoting cell proliferation while inhibiting apoptosis. *BRAF* V600E-mutated BTC has been more recently associated with higher tumor-node-metastasis (TNM) stage, resistance to systemic chemotherapy, aggressive clinical course and worse survival [76, 77], although early studies suggested no correlation between survival and *BRAF* mutations. Robertson and colleagues [77] identified, by using immunohistochemistry (IHC), *BRAF* mutations in 7.4% of iCCA patients, with a longer OS in wild-type patients than in *BRAF*-mutated subjects (37.3 and 13.5 months, respectively).

Activating mutations in *EGFR* were described in two small studies in 13.6% (3/22) and 20% (3/15) of cases [78, 79]. Prognostically, *EGFR* expression has been found to be a negative predictor of OS in CCA [63, 80], making this an attractive target for drug intervention [81]. However, no clinical trial demonstrated up to now the activity of anti-EGFR agents in iCCA.

RAS proteins are small GTPases that function as switch molecules by coupling cell membrane growth factor receptors [82]. Gain-of-function KRAS variants impair the GTPase activity thus leading the RAS protein in a constitutive active status. *KRAS* activating mutations have been identified in 8–54% of patients with iCCA, and generally higher incidence of *KRAS* mutations are associated with increased tumor stage and poor prognosis [75].

Mutations leading to inactivation of tumor suppressor genes have been described to have prognostic significance. Loss of function mutations of the *TP53* gene have been reported in about 20% of iCCA and are correlated to a worse prognosis in iCCA. *BAP1* mutations were identified exclusively in patients with iCCA and not in other CCA subtypes [83] representing one of the most frequent mutations found in iCCA with about 20% of the overall cases carrying variants in this gene [27].

*NTRK* fusions are reported in less than 1% of CCA patients [84]. In *NTRK* gene fusion events, the resulting fusion gene encodes a protein containing the N-terminus of the fusion partner joined to the C-terminus of the tropomyosin receptor kinase (TRK) protein, including the catalytic tyrosine kinase domain. Most *NTRK* gene fusions contain a 5’ partner gene sequence encoding dimerization domains. These domains regulate the activity of the generated tyrosine kinase, thus conferring a ligand-independent oncogenic potential through uninterrupted downstream signaling messages, promoting cell proliferation and survival [85, 86].

Mutations in other genes such as axin 1 (*AXIN1*, 41%), APC regulator of WNT signaling pathway (*APC*, 13%), cadherin 1 (*CDH1*, 11%), catenin beta 1 (*CTNNB1*, 8%), *BRCA1* (0.4%), and *BRCA2* (2.7%) have also been reported in iCCA [87, 88].

### eCCA

Molecular characterization of eCCA was hindered by the small number of eCCA cases included in international cancer genome projects and by the strong heterogeneity of the cohorts examined. Consequently, a reliable consensus on targetable genes to provide therapeutic options has not yet been established contributing to the current lack of targeted therapies for eCCA patients [89].

The first large comprehensive genomic profiling study of eCCA samples analyzed 99 cases and established that 99% of the samples harboured at least one genomic alteration, while 83% of samples harboured at least one clinically relevant genomic alteration. The most common clinically relevant genomic alterations were *KRAS* (43%), *ERBB2* (9%), phosphatase and tensin homolog (*PTEN*, 7%), ATM serine/threonine kinase (*ATM*, 6%), neurofibromin 1 (*NF1*, 6%), cyclin D1 (*CCND1*), F-box and WD repeat domain containing 7 (*FBXW7*), GNAS complex locus (*GNAS*), MDM2 proto-oncogene (*MDM2*) and *NRAS* (all at 5% frequency) (Table 1). In one out of 99 patients (1%), alterations were found in *BRAF*, *BRCA2*, cyclin dependent kinase 4 (*CDK4*), *CDK6*, *FGFR1*, *FGFR3*, patched 1 (*PTCH1*), RAF or serine/threonine kinase 11 (*STK11*). The most frequently non-actionable alterations included *TP53* (45%), cyclin dependent kinase inhibitor 2A (*CDKN2A*, 28%), cyclin dependent kinase inhibitor 2B (*CDKN2B*, 15%), SMAD family member 4 (*SMAD4*, 15%) and *ARID1A* (13%) [90]. High rate of *TP53* alterations (68%) was further observed in a Chinese cohort of patients with eCCA (*n* = 80) [91].

The frequency of *ERBB2* alterations was 9% (6/9 alterations were base substitution/insertion mutations and 3/9 were amplifications). No *IDH1/2* mutations or *FGFR2* gene fusions were identified in this cohort of patients [90]. Similar frequency was observed for alterations in *BRCA2* and *BRCA1* (2.6% for *BRCA2 vs.* 2.1% for *BRCA1*) [92].

In the study by Nakamura and colleagues [18], ATPase Na^+^/K^+^ Transporting Subunit Beta 1/Protein Kinase CAMP-Activated Catalytic Subunit Alpha (*ATP1B1*-*PRKACA*) and *ATP1B1*-*PRKACB* fusions were exclusively detected in a single eCCA case. HER2 overexpression was found in 5–9% of eCCA [93].

Recently, in a study including an international multicenter dataset of 189 patients with eCCA, genes such as *KRAS* (36.7%), *TP53* (34.7%), *ARID1A* (14.0%) and *SMAD4* (10.7%) were found frequently mutated. Furthermore, 25% of tumors analyzed in this study have targetable genomic alterations in several genes including *EGFR*, *ERBB2*, *BRCA1/2*, *IDH1/2*, *CDK4*, *BRAF*, *PIK3CA*, *MDM2* and *NRAS*. Recurrent chromosomal amplifications were also observed in YEATS domain containing 4 (*YEATS4*, 6.0%), *MDM2* (4.7%), cyclin E1 (*CCNE1*, 2.7%), *CDK4* (1.3%) and *ERBB2* (1.3%) [94].

In the same study, unsupervised clustering of whole-genome expression data revealed 4 distinct molecular groups of eCCA with distinct oncogenic signatures and histological subtypes. The “Metabolic class” (19%) was defined by a hepatocyte-like phenotype with activation of the transcription factor hepatocyte nuclear factor 4 alpha (HNF4A) and disruption of bile acid and fatty acid metabolism. Tumors classified within the “Proliferation class” (23%), more common in patients with dCCA, were characterized by the overexpression of MYC proto-oncogene (*MYC*) targets, *ERBB2* mutations/amplifications and activation of mTOR signaling. The “Mesenchymal class”, accounting for 47% of cases, was characterized by signatures of EMT, aberrant transforming growth factor beta 1 (TGFb) signaling and poor OS [94]. Finally, the eCCA “Immune class” (11%) defined a population that could better respond to immune checkpoint inhibitors showing a higher lymphocyte infiltration and overexpression of programmed cell death protein 1/programmed death-ligand 1 (PD-1/PD-L1) [94].

## Technologies for genomic profiling of CCA

As above described, genomic profiling of CCA might allow identifying actionable genetic alterations that can guide treatment decision and offer possibility of treatment with targeted therapies.

Actionable genomic alterations in CCA include single nucleotide variants (SNVs), insertions and deletions (indels), gene amplifications and chromosomal rearrangements leading to gene fusions, which require different techniques for their detection in tumor specimens, including IHC, FISH or various strategies involving amplification of the DNA or RNA sequence by polymerase chain reaction (PCR) [71, 95–101].

In clinical practice, IHC is being used as a screening method to detect fusions ALK receptor tyrosine kinase (ALK) or to select cases with suspected fusions (ROS1, NTRK) that are subsequently confirmed with ortogonal techniques. However, no IHC method has yet been validated for the detection of FGFR2 fusions [46]. Therefore, IHC does not allow a screening for the iCCA cases with suspect FGFR2 fusions.

The most common method to detect gene fusions is break-apart FISH assay performed on formalin fixed paraffin embedded (FFPE) tumors. This approach has shown a good sensitivity and specificity to detect FGFR2 fusions [102]. However, it must be emphasized that approximatively 50% of all FGFR2 rearrangements in iCCA are intrachromosomal, and FISH may not discriminate cases where the distance between the 5’ and 3’ probes after rearrangement remains too short, thus resulting in possible false negative results [103]. Comprehensive genomic profiling and clinical outcomes in patients with fibroblast growth factor receptor rearrangement-positive CCA treated with pemigatinib in the FIGHT-202 trial [104].

PCR-based assays provide the greatest versatility in detecting mutations; in particular DNA-based quantitative PCR can be used for specific deletion mutations and SNVs in exon sequences [105–107]. Furthermore, reverse transcriptase-PCR (RT-PCR) is a fast and sensitive method able to detect transcribed gene fusions, where both merging partners and breakpoint location are known [105, 108]. Unfortunately, the limit of this technology is the necessity of the presence of a previously described sequence of the region in which the fusion occurs. In particular, RT-PCR has several limitations for *FGFR2* fusion testing in CCA, since a large number of potential *FGFR2* fusion partners are mostly still unknown.

The alternative approach to genomic profiling of CCA is NGS, a cost-effective technology that allows the identification of different types of genomic alterations in multiple genes in a single analysis [109].

NGS-based targeted sequencing may differ for the initial source material (e.g., FFPE tumor tissue or peripheral whole blood), the technologies for library preparation [hybrid capture based, amplicon based, or anchored multiplex PCR (AMP)], and the nucleic acids tested for fusion detection (DNA and/or RNA).

In routine clinical practice, the preferred source for genomic profiling is tumor tissue, generally obtained through invasive procedures such as resection or biopsies.

The main starting materials for genomic profiling platforms based on NGS are DNA or RNA, and three strategies can be used for target enrichment, hybrid capture [110–112], Memorial Sloan Kettering integrated mutation profiling of actionable cancer targets (MSKIMPACT) [95, 113]; amplicon-based and AMP approaches (e.g., Oncomine™ Dx Target Test [114] and Archer FusionPlex Solid Tumor Panel [115]).

Tumor-derived DNA is more stable as compared with RNA. However, when testing fusions, it must be underlined that the presence of intronic regions might limit the sensitivity of the assay, depending also from the method used for the preparation of the library.

Target enrichment via hybrid capture is achieved by using gene-specific hybridization probes to select the desired target sequences from shotgun genomic DNA libraries (DNA-based method) [110] or from libraries of expressed transcripts (RNA-based method) [116]. Hybrid capture-based NGS has been used for comprehensive genomic profiling of CCA with large gene panels (> 400 genes) [25, 26], able to detect all different types of genetic alterations including base substitutions, indels, rearrangements, gene amplifications, MSI and TMB.

Amplicon-based library preparation has the advantage to require a lower amount of input nucleic acid. The possibility to detect fusions at DNA levels with the amplicon-based technology is significantly limited by the presence of intronic regions. This issue can be overcome by RNA sequencing. However, the presence of > 100 *FGFR2* fusion partners in iCCA represents a significant limit for the clinical sensitivity of the assay, since primers are available only for known fusions. Newer NGS panels include a 5’-3’ imbalance assay that can suggest the presence of a fusion not covered by the primers included in the assay. This approach still needs validation on large cohort of cases.

RNA-based AMP is particularly useful to detect fusions in different genes [117] especially genes that have a large number of known and unknown fusion partners, as in the case of *FGFR2* [25]. This approach combining the use of universal and gene-specific primers in multiplexed assays, allows the simultaneous detection of any known or unknown 5’ or 3’ prime fusion partner of multiple targets [118].

Although different techniques can be used for genomic profiling of CCA, the relative low amount of tumor tissue available in most cases and the high number of actionable mutations that offer potential for therapeutic intervention, make NGS the preferred testing method in this disease. In this respect, the FoundationOne^®^ CDx test (a large-scale genomic profiling assay targeting up to 324 genes) was approved by FDA as a companion diagnostic test for pemigatinib therapy in CCA patients with FGFR2 fusions or other rearrangements [112]. Furthermore, the European Society For Medical Oncology (ESMO) Precision Medicine Working Group issued recommendations for the use of NGS in patients with specific types of advanced cancer, including CCA. The group recommended to use targeted multigene NGS-based panels for detecting in CCA level I actionable alterations, according to the ESMO Scale for Clinical Actionability of molecular Targets (ESCAT). ESCAT level I genomic alterations are biomarkers validated in clinical trials and ready for clinical use, which include *IDH1* mutations, *FGFR2* and *NTRK* fusions, and MSI-high in CCA [119].

## CCA molecular profiling by cell-free DNA testing

Tumor tissue sampling is sometime unfeasible, especially in patients with advanced disease, for different reasons including the risk and inaccessibility of tumor. In addition, in some cases tissue can result insufficient or inadequate for biomarker analysis. This is common in the tumors from the biliary tract and in particular for primary CCA, in which the tumor is not easily accessible. In fact, a recent study on 149 samples from 104 patients with advanced BTC has shown a failure of analysis of tissue biopsies in 26.8% of cases, for the inadequate tumor content [120]. In patients with no tissue available for genomic profiling or inadequate material, the sequencing of cell-free DNA (cfDNA) might represent a valuable alternative. cfDNA testing presents several advantages over tissue testing, including the low invasiveness and the capability to better recapitulate the molecular heterogeneity of the disease and its dynamic evolution. However, the amount of cfDNA isolated from peripheral plasma is limited, and only a fraction of the cfDNA derives from tumor cells (circulating tumor DNA, ctDNA) thus limiting the sensitivity of the test.

PCR- and NGS-based methods are the two dominant approaches in the field of cfDNA analysis [121]. In particular, new NGS techniques with improved sensitivity have been recently developed to overcome the limited sensitivity of standard targeted sequencing approaches [122].

In the past few years, several studies have demonstrated the feasible and the clinical utility of cfDNA-based molecular profiling in CCA patients. A multiplex digital PCR assay was used to screen for 31 mutations in *KRAS*, *NRAS*, *BRAF*, and *PIK3CA* genes in plasma samples from CCA patients [123]. The assay correctly classified samples with known mutational status based on tissue testing. However, the applicability of this assay for CCA may be limited due to the low frequency of *KRAS*, *BRAF*, and *PIK3CA* mutations [124].

In a large study by Mody and co-workers [125], 124 BTC patients (70% with iCCA) were enrolled to evaluate the utility of cfDNA analysis using a 73-gene NGS panel. At least one therapeutically relevant genomic alteration was observed in 55% of patients, and 21% of patients had one of the most frequently occurring actionable alterations, including *BRAF*, *FGFR2* and *IDH1* mutations, *ERBB2* amplification, *FGFR2* fusions. Genomic alterations identified in iCCA were found different from those found in eCCA or gallbladder cancer. Furthermore, therapeutically relevant alterations were more frequent in iCCA than in eCCA. In particular, *FGFR2* gene alterations were more frequent in iCCA patients, while ERBB2 alterations were detected exclusively in eCCA [125].

In BTC patients in whom both ctDNA and tissue-DNA were sequenced with NGS, the overall concordance rates for *TP53*, *KRAS* and *PIK3CA* genomic alterations were higher between ctDNA and tissue-DNA obtained from metastatic site, than between ctDNA and primary tumor DNA (78% *vs.* 65% for *TP53*, 100% *vs.* 74% for *KRAS*, and 100% *vs.* 87% for *PIK3CA*) [126]. These data suggest that liquid biopsy might better recapitulate the genomic profile of metastatic disease. Interestingly, among 80 patients who received systemic treatment, the molecularly matched therapeutic regimens based on ctDNA and/or tissue-DNA molecular profiling showed a significantly longer progression-free survival and higher disease control rate than unmatched regimens [126].

In the study by Ettrich et al. [127], tumor tissue and corresponding ctDNA samples were collected from patients with CCA at predefined time points (prior to treatment initiation, 1.7 ± 0.8 months after treatment initiation, and progression) and were subjected to deep sequencing of 15 genes frequently mutated in CCA. The results showed that the mutational profile of the 23 available blood-tumor pairs was concordant for 74% of patients, with a higher concordance rate between mutations in tumor tissue and ctDNA in iCCA (92%) *vs.* eCCA (55%) [127].

Moreover, to evaluate the utility of ctDNA genotyping in patients with gastrointestinal cancer, Nakamura and colleagues [128] compared trial enrollment in ctDNA-based screening study, called GOZILA, which used ctDNA-based NGS screening, *vs.* gastrointestinal (GI) ctDNA-based screen study (GI-SCREEN) that employed tissue for genotyping. The ctDNA approach significantly shortened the screening duration (11 days *vs.* 33 days, *P* < 0.0001) and improved the trial enrollment rate (9.5% *vs.* 4.1%, *P* < 0.0001) without compromising treatment efficacy compared to tissue genotyping. Importantly, genetic alterations were identified in > 90% of CCA patients, thus confirming a relatively high sensitivity of liquid biopsy in this specific subset of patients.

The usefulness of ctDNA for disease monitoring and detection of acquired resistance during the targeted therapy was demonstrated by Goyal and colleagues [129] in a phase 2 study of the FGFR inhibitor infigratinib (BGJ398). Three patients with advanced iCCA underwent serial ctDNA testing at enrollment and after radiologic progression. ctDNA analysis demonstrated de novo point mutations in the *FGFR2* gene possibly conferring resistance to BGJ398 at the time of testing upon experiencing disease progression [129]. Similar data were reported by Silverman and co-workers in the FIGHT-202 trial of pemigatinib in FGFR-rearranged CCA by using either tissue or liquid biopsy testing [25]. Based on these studies, ctDNA sequencing could potentially improve the clinical management of CCA patients, though the low sensitivity in the detection of translocations, in particular in the *FGFR2* gene, still poses a challenge and needs further experimental and clinical validations to demonstrate the clinical utility of ctDNA profiling in the management of CCA.

## Clinical trials

The identification of driver mutations, which promote tumor growth, survival and progression, has allowed the development of specific inhibitors capable of selectively blocking the altered pathways in neoplastic cells. This approach, defined as “target therapy”, requires the identification of biomarkers (i.e., driver genomic alterations) and specific matched drugs, thus realising what is now called precision medicine. In this respect, CCA represents today one of the main tumors in which it has been possible to identify possible biomarkers for matched therapies in a significant fraction of cases. Selected clinical trials investigating the most promising molecular targets and matched drugs in CCA are reported in Table 2. In particular, 17 clinical trials, targeting 4 different genomic alterations (*IDH1/2*, *FGFR*, *NTRK*, and *BRAF*) with 13 distinct drugs has been identified. Of the 17 trials, 12 are still ongoing and 5 had final results that are summarized in Table 2.

**Table 2.** Recent and ongoing clinical trials investigating agents targeting the key driver mutations in CCA

**Target**	**Targeted agents**	**Study**	**Patient population (*n*)**	**Status**	**Results**	**NCT number**
IDH1/2	Ivosidenib	Phase 1, multicenter, open-label	73	Active, not recruiting	Ongoing	NCT02073994
		Phase 3, multicenter, (ClarIDHy)	185	Active, not recruiting	mPFS = 2.7 mos (ivosidenib) *vs.* mPFS = 1.4 mos (placebo)	NCT02989857
	FT2012	Phase 1/2	200 (estimated)	Active, not recruiting	Ongoing	NCT03684811
	BAY1436032	Phase 1, open-label, non-randomized, multicenter	81	Active, not recruiting	Ongoing	NCT02746081
FGFR2	Pemigatinib	Phase 2, open-label, single-arm, multicenter study (FIGHT-202)	146	Active, not recruiting	ORR = 35.5%; PFS = 6.9 mos; DoR = 7.5 mos (interim results for 107 pts)	NCT02924376
		Phase 3, open-label, randomized, active-controlled, multicenter (FIGHT-302)	432 (estimated)	Recruiting	Ongoing	NCT03656536
	Infigratinib	Phase 2, single arm	160 (estimated)	Recruiting	ORR = 14.8%; DCR = 75.4%; mPFS = 5.8 mos (interim results for 61 pts)	NCT02150967
		Phase 3 multicenter, open-label, randomized (The PROOF Trial)	384 (estimated)	Recruiting	Ongoing	NCT03773302
	Derazantinib	Phase 2, open-label, single-arm study	143 (estimated)	Recruiting	Ongoing	NCT03230318
	Futinatinib	Phase 1/2	386	Active, not recruiting	ORR = 37.3%; DCR = 82.1%; mPFS = 7.2 mos; mDoR = 6.2 mos (interim results)	NCT02052778
		Phase 3, open-label, randomized (FOENIX-CCA3)	216 (estimated)	Recruiting	Ongoing	NCT04093362
	Erdatifinib	Phase 2	35	Active, not recruiting	Ongoing	NCT02699606
	Debio-1347	Phase 2 basket (FUZE Clinical Trial)	63	Active, not recruiting	Ongoing	NCT03834220
	Ponatinib	Phase 2	45 (estimated)	Recruiting	Ongoing	NCT02272998
NTRK	Entrectinib	Phase 2 basket, open-label, multicenter, global (STARTRK-2)	700 (estimated)	Recruiting	Ongoing	NCT02568267
	Larotrectinib	Phase 2 basket (NAVIGATE)	203 (estimated)	Recruiting	Ongoing	NCT02576431
BRAF	Dabrafenib + trametinib	Phase 2, open-label	206	Active, not recruiting	ORR = 47%; mDoR ≥6 mos in the 54% of responders; PFS = 7.2 mos; OS = 11.3 mos (interim results for 43 pts)	NCT02034110

mPFS: median progression-free survival; DCR: disease control rate; mos: months; ORR: overall response rate; DoR: duration of response; mDoR: median duration of response; pts: patients

A number of ongoing clinical trials are currently investigating IDH1/2 inhibitors. In a phase 1 clinical trial (NCT02073994), 73 previously treated patients with *IDH1* mutant CCA were treated with ivosidenib. In this trial, ivosidenib showed promising clinical activity with a mPFS of 3.8 months (95% CI 3.6–7.3 months), 6-month progression-free survival (PFS) of 40.1% (28.4–51.6%), 12-month PFS of 21.8% (12.3–33.0%), and a mOS of 13.8 months (11.1–29.3 months) [130].

In the multicentre phase 3 ClarIDHy study (NCT02989857), patients with advanced pretreated IDH1-mutant CCA were randomly assigned to receive ivosidenib (*n* = 124) or placebo (*n* = 61). This study demonstrated a significant improvement of mPFS with ivosidenib compared to placebo (median 2.7 months *vs.* 1.4 months) [131]. Based on these findings, the FDA has recently granted a priority review to the application for ivosidenib as a new treatment option for patients with previously treated, *IDH1*-mutant CCA.

Other IDH1/2 inhibitors such as FT2012 (NCT03684811) or BAY1436032 (NCT02746081) are currently tested in *IDH1/2* mutant advanced solid tumors, including CCA.

Furthermore, since the homologous repair (HR) deficiency conferred by *IDH1/2* mutations is known to make cells more sensitive to Poli ADP-ribosio polimerasi (PARP) inhibition, the PARP inhibitor olaparib is currently tested in the phase 2 trial of *IDH1/2* mutant relapsed solid tumors, including CCA (NCT03212274).

However, in the study by Eder and coworkers, none of four patients with *IDH1/2*-mutant CCA derived clinical benefit from Olaparib [132]. Interestingly, the CCA patients enrolled in this trial had multiple other mutations in addition to the *IDH* variants, including those in chromatin modulators or putative signal transduction drivers, which might have affected the activity of olaparib.

Several selective and non-selective inhibitors of FGFR are currently being investigated in different clinical trials in CCA. The results of some of these trials have completely changed the diagnostic and therapeutic approach to CCA. The anti-tumour activity of pemigatinib, a selective inhibitor of FGFR 1–3, was evaluated in the phase 2 study FIGHT-202 (NCT02924376) in 146 patients with previously treated metastatic CCA, with and without FGFR2 alterations. In particular, 107 patients harboured *FGFR2* fusions or rearrangements, 20 other fibroblast growth factor (FGF)/FGFR alterations, and 18 had no FGF/FGFR alterations. For patients with *FGFR2* fusions or rearrangements, mPFS was 6.9 months (95% CI 6.2–9.6 months); 2.1 months (95% 1.2–4.9 months) for patients with other FGF/FGFR alterations, and 1.7 months (95% CI 1.3–1.8 months) for patients without *FGFR* alterations [131]. Based on these results, pemigatinib was approved by FDA for the second-line treatment of metastatic CCA patients with *FGFR* rearrangements or fusions. The phase 3 study FIGHT-302 (NCT03656536) is ongoing to evaluate the efficacy and safety of pemigatinib *vs.* gemcitabine plus cisplatin in the first-line treatment of patients with metastatic CCA with *FGFR2* rearrangements [133].

Interestingly, in patients carrying *FGFR2* fusions and treated with the FGFR inhibitor pemigatinib, it was found a statistically significant inverse correlation between the presence of genomic alterations in any tumor suppressor genes and PFS [25]. In fact, patients with altered tumor suppressor genes had a shorter PFS as compared with unaltered patients. A similar correlation was found for specific alterations of *TP53*, *CDKN2A/B* and *PBRM1*, with a trend for *BAP1*.

Sixty-one patients with pre-treated metastatic CCA carrying *FGFR2* alterations were enrolled in a phase 2 (NCT02150967), single arm clinical trial investigating the efficacy of BGJ398, a potent and selective ATP-competitive inhibitor for FGFR1/2/3. The results showed an ORR of 14.8%, DCR was 75.4% and estimated mPFS was 5.8 months [134].

Treatment with derazantinib produced an ORR of 20.7%, a DCR of 82.8%, and a mPFS of 5.7 months in previously treated patients with advanced iCCA and *FGFR2* fusions (NCT01752920) [135]. A pivotal trial of derazantinib in iCCA is ongoing in subjects with inoperable or advanced iCCA whose tumors harbor *FGFR2* gene fusions or *FGFR2* gene mutations or amplifications and who received at least one prior regimen of systemic therapy (NCT03230318).

Other phases 3 studies are ongoing in patients with untreated advanced CCA harbouring *FGFR2* rearrangements to evaluate the efficacy of infigratinib (NCT03773302) and futibatinib (NCT04093362) *vs.* gemcitabine plus cisplatin chemotherapy in first-line treatment.

Additional studies of erdatifinib (NTC02699606), Debio-1347 (NTC03834220), ponatinib (NTC02272998) are ongoing.

FGFR inhibitors have shown encouraging results in different clinical trials. However, mechanisms of acquired resistance with the occurrence of secondary mutations have been reported to reduce the duration of the response. In particular, in 3 patients with *FGFR2* fusion-positive CCA treated with BGJ398, the occurrence of a secondary mutation in the *FGFR2* kinase domains in one patient and multiple *FGFR2* mutations in the remaining two patients were observed. Interestingly, the mutation *FGFR2* p.V564F was identified in all patients, suggesting a relevant role of this genomic alteration in the resistance to anti-FGFR agents [129]. Similar findings were reported in patients progressing on treatment with pemigatinib [25]. In fact, every patient who progressed on pemigatinib showed at least one acquired mutation in the *FGFR2* kinase domain, spanning five amino acid residues (p.N550H/K, p.E566A, p.K660M, p.L617V, p.K641R) [25].

Additional studies will be required to determine the impact of these kinase domain mutations on FGFR2 fusion protein as well as it will be important to establish the extent to which preexisting *FGFR2* mutations affect the time to treatment failure, as observed in non-small cell lung cancer carrying *EGFR*-mutation.

A recent study in patients with fusion-positive iCCA who progressed on BGJ398 or Debio1347 revealed that treatment with the ATP-competitive FGFR inhibitor futibatinib might overcome the acquired resistance to FGFR reversible inhibitors (NCT02052778) [136]. Additional studies in a larger population of patients are needed to confirm these findings.

Although *NTRK* fusions are rare in CCA, the robust and durable response of NTRK inhibitors in patients with advanced solid tumors harbouring *NTRK* gene fusions led to add these targeted agents to the therapeutic options of CCA. Particularly, two potent TRK inhibitors, entrectinib and larotrectinib, have recently emerged as novel therapeutic options in *NTRK* fusion-positive malignancies [137].

Entrectinib is currently been assessing in an open-label, multicenter, global phase 2 basket study (STARTRK-2) for the treatment of patients with solid tumors that harbor an *NTRK1/2/3*, *ROS1*, or *ALK* gene fusion (NCT02568267). No data on CCA are available so far for larotrectinib, which is currently being assessed as monotherapy in the phase 2 NAVIGATE basket trial on *NTRK* fusion-positive malignancies, including advanced CCAs (NCT02576431). Importantly, both drugs have been approved as agnostic therapies for patients with *NTRK* fusion-positive cancer, independently from their histology.

The most common *BRAF* mutation occurring in approximately 5% of iCCA patients is the V600E. The combination of dabrafenib (a BRAF inhibitor) and trametinib (a MEK inhibitor) has shown activity in several *BRAF* V600E-mutated cancers [138].

The Rare Oncology Agnostic Research (ROAR) basket trial (NCT02034110), was designed to determine as primary study endpoint the ORR of dabrafenib in combination with trametinib in patients with rare *BRAF* V600E-mutated cancers, including 43 patients with advanced *BRAF* V600E mutated BTC (iCCA patients, *n* = 39). The ORR was 47% (95% CI 31–62%), while PFS was 7.2 months and OS was 11.3 months. These results, coupled with few side effects, support consideration of dabrafenib plus trametinib combination treatment in patients with *BRAF* V600E-mutated BTC [139].

## Conclusion

While incidence of CCA is rising, the prognosis of advanced CCA patients remains dismal, due to the relative lack of efficacy of conventional treatments. In this scenario, a greater understanding of the pathogenesis and the genetic and molecular characteristics of CCA is essential in order to improve the diagnosis and therapy of this aggressive tumor. Experimental studies of lineage tracing, in animal models, have indicated that CCAs may derive from either biliary progenitor cells or mature cholangiocytes [8]. However, controversy exists and future research will need to clarify the similarities between experimental models and the human disease. In this regard, the analysis at the single cell level could provide critical information on this issue and clarify the role of the microenvironment in shaping different tumour phenotypes.

Studies of genomic profiling revealed the molecular heterogeneity and complexity of CCA, which can be classified in different entities based on the specific pattern of genomic alterations in addition to its histological and pathological features. Notably, level I actionable genomic alterations, which offer possibility to treat patients with matched therapies already approved or in advanced phase of clinical development, can be found in up to 40% of CCA [119]. After lung adenocarcinoma, CCA is currently the second tumor type in which a significant fraction of patients might benefit treatment with targeted therapies. These findings open new possibilities of therapy in a disease with dismal prognosis and relative resistance to chemotherapy.

The relative high number of genomic alterations to test, the complexity of these alterations with specific regard to gene fusions, the relatively low amount of tissue available for the majority of patients in advanced stage, make NGS as the preferred approach for genomic profiling of CCA [119]. Different methods for library preparation starting from either DNA or RNA are available, and each of these has advantages and limits [41]. While the technology is evolving, the capability of NGS testing in many European countries is still extremely limited [140]. In addition, CCA is a relatively rare disease for which there is a certainly lower level of attention than lung cancer by the health authorities and the oncology community. Education and information of all stakeholders will have a relevant role to make possible the access of CCA patients to NGS testing and, consequently, targeted therapies.

Comprehensive genomic profiling of CCA is revealing complex patterns with co-mutations that can possibly affect response to targeted agents. In particular, the presence of genetic alterations in tumor suppressor genes was found to correlate with a shorter PFS in CCA patients with FGFR2 fusions treated with pemigatinib [25]. These findings are in agreement with our previous results in lung cancer suggesting that the presence of specific co-mutations might reduce the efficacy of EGFR tyrosine kinase inhibitors in EGFR-mutant patients [141]. While these observations need further confirmation in larger cohorts of patients, they raise the importance of comprehensive genomic profiling for a better understanding of the biology of the tumor and to improve stratification of patients. If different therapeutic options will become available for first line treatment of CCA patients (chemotherapy, target therapy, immunotherapy), a comprehensive profile of the genomic alterations of the tumor of each individual patient might better support the choice of the therapeutic strategy.

Comprehensive genomic profiling might also help in assessing the clonality of the identified mutations. In this respect, most studies of genomic profiling of CCA did not report information on the clonal or sub-clonal origin of the identified actionable mutations. In addition, evidence suggests that some CCA carry a considerable portion of sub-clonal mutations [142]. We might expect that tumors with sub-clonal driver alterations might not respond to matched therapies. In addition, the presence of sub-clones carrying resistance mutations might lead to early resistance to targeted therapy.

Increasing evidence suggests that liquid biopsy, and in particular NGS testing of cfDNA, can have clinical applications in patients with CCA. In addition to genomic profiling in patients with no tissue available, cfDNA testing might provide a minimally invasive method to allow monitoring the response to therapy, disease relapse, progression and clonal evolution of the disease and identifying mechanisms of resistance. The availability of new FGFR inhibitors might in the future allow the alternation of different inhibitors based on the results of cfDNA testing. This strategy has proven efficacious in other tumor types and could be explored in CCA as well.

In conclusion, CCA is a relatively rare and aggressive cancer in which it has been possible to identify a number of genomic alterations for which targeted therapies are available. Therefore, genomic profiling should be mandatory in all patients with advanced CCA in order to provide the most appropriate therapeutic strategy. Comprehensive genomic profiling of CCA will also contribute to increase the knowledge on the biology of this disease and to better define the impact of the genomic landscape on the response to targeted agents. Due to its clinical and biological characteristics, the CCA can therefore become a paradigm for the application of precision oncology in clinical practice, representing a study model that can then be exported to other cancers.

## Abbreviations

2-HG: d-2-hydroxyglutarate
AMP: anchored multiplex polymerase chain reaction
ARID1A: AT-rich interaction domain 1A
BAP1: BReast CAncer gene 1 associated protein 1
BRAF: B-Raf proto-oncogene
BRCA1: BReast CAncer gene 1
BTCs: biliary tract cancers
CCA: cholangiocarcinoma
CDK4: cyclin dependent kinase 4
CDKN2A: cyclin dependent kinase inhibitor 2A
cfDNA: cell-free DNA
ctDNA: circulating tumor DNA
dCCA: distal cholangiocarcinoma
DCR: disease control rate
DoR: duration of response
eCCA: extrahepatic cholangiocarcinoma
EGFR: epidermal growth factor receptor
ERBB2: erb-b2 receptor tyrosine kinase 2
ERK: extracellular signal-regulated kinase
FGFR2: fibroblast growth factor receptor 2
FISH: fluorescence in situ hybridization
GTPase: guanosine triphosphate hydrolase
iCCA: intrahepatic cholangiocarcinoma
IDH: isocitrate dehydrogenase
IHC: immunohistochemistry
KRAS: KRAS proto-oncogene
MAPK: mitogen-activated protein kinase
MDM2: MDM2 proto-oncogene
mDoR: median duration of response
MEK: mitogen-activated extracellular signal-regulated kinase kinase
mPFS: median progression-free survival
MSI: microsatellite instability
NGS: next-generation sequencing
NTRK: neurotrophic receptor tyrosine kinase
ORR: overall response rate
OS: overall survival
PBRM1: polybromo 1
pCCA: perihilar cholangiocarcinoma
PCR: polymerase chain reaction
PFS: progression-free survival
PIK3CA: phosphatidylinositol-4,5-bisphosphate 3-kinase catalytic subunit alpha
RAF: Raf-1 proto-oncogene, serine/threonine kinase
RAS: rat sarcoma virus
ROS1: ROS proto-oncogene 1, receptor tyrosine kinase
TP53: tumor protein p53
TRK: tropomyosin receptor kinase

## Declarations

### Copyright

© The Author(s) 2022.
